# Rabies in equids in Sudan

**DOI:** 10.4102/ojvr.v91i1.2181

**Published:** 2024-09-26

**Authors:** Yahia H. Ali, Tenzeil A.G. Mohieddeen, Muaz M. Abdellatif, Baraa Mohammed Ahmed, Intisar K. Saeed, Husham M. Attaalfadeel, Amani A. Ali

**Affiliations:** 1 Department of Biology, College of Science and Arts, Rafha, Northern Border University, Arar, Saudi Arabia; 2 Virology Department, Central Veterinary Research Laboratory, Khartoum, Sudan; 3 Department of Mathematics, College of Science and Arts, Rafha, Northern Border University, Arar, Saudi Arabia; 4 Department of Economics, Faculty of Economic and Social Sciences, The Holy Quran University, Omduman, Sudan; 5 Department of Pharmacology and Toxicology, College of Pharmacy, Rafha, Northern Border University, Arar, Saudi Arabia

**Keywords:** rabies, equine, fluorescent antibody test, reverse transcriptase polymerase chain reaction, Sudan

## Abstract

Rabies is endemic in Sudan with continuing outbreaks occurring annually, the most common animals affected are dogs, followed by goats and equids. This work focused on equid rabies, to elucidate the current situation of the disease through analysis of reports of equid rabies outbreaks in Sudan during 2010–2022 supported by laboratory confirmation of the disease. During the study period, 66 animals were affected during 35 equid rabies outbreaks. The highest incidences were found in Al Gezira (30.3%), followed by Darfur (24.2%) and Kordofan (15.2%). The highest incidence rate was observed during 2018 (33.3%), followed by 2015 (16.7%). Within seasons, the highest incidence rate was reported during October – December (33.3%), followed by July – September (30.3%). Chi-square analysis revealed a significant correlation between rabid animals and year, season, and state. Wald statistics demonstrated that year and season had a significant association with the disease. Virus antigen was identified (72.2%) in brain tissues using the fluorescent antibody test. Viral nucleic acid was amplified (*n* = 6) with a reverse transcriptase polymerase chain reaction assay.

**Contribution:**

As equids are kept in close contact with humans and other animals in the country, according to the present investigation, equid rabies in Sudan is a potential public health concern, emphasising the importance of implementing effective control measures.

## Introduction

Equids are very important animals in Sudan, as they are used for riding, carrying goods and in remote areas they are used for public transport; therefore, they are considered of economic importance. The estimated equid population worldwide is 114 million, 97% of which are kept in developing countries, and represent 59 million horses, 44 million donkeys and 11 million mules (Barrandeguy & Carossino 2018; Getachew, Burden & Wernery 2016). The estimated number of horses in Sudan in 2020 was 793 252, while that of donkeys was 7 631 698 (Anon 2020). Rabies is one of the most life-threatening diseases in humans and animals (Singh et al. 2017). The virus is classified as belonging to the genus *Lyssavirus* of the family *Rhabdoviridae.* Rabies is one of the most fatal neurological zoonotic diseases (Fisher, Streicker & Schnell 2018; Susan 2023). It affects all mammals, both wild and domesticated; dogs and cats are the most commonly affected species; however other domestic animals are susceptible to the disease with variable incidences (Krebs et al. 2001). It was thought to be one of the most reported zoonotic viral diseases in donkeys (Banyard et al. 2013). In Algeria, donkeys are the third most common rabies-affected mammal (Banyard et al. 2013).

Equids as well as other domestic animals are susceptible to rabies and reports about the existence of equid rabies in different parts of Africa have been published (Khalafalla & Ali 2022). Many reports from Eastern Africa documented the presence of rabies in donkeys and horses in Ethiopia (Digafe, Kifelew & Mechesso 2015; Gizachew et al. 2012; Stringer et al. 2017). The disease was described in equids in Kenya (Bitek et al. 2018), in Western Africa in Burkina Faso (Minoungou et al. 2021), South Africa (Koeppel, Van Schalkwyk & Thompson 2022) and in North Africa, in Tunisia (Kalthoum et al. 2021). In Asia many reports of rabies in equids were published, in mules in Pakistan (Numan et al. 2011), in horses and donkeys in Jordan (Faizee et al. 2012) and in China (Feng et al. 2016). In Latin America, rabies cases have been documented in Brazil (Coelho et al. 2022; Oliveira et al. 2022; Pimentel et al. 2022; Schwarz et al. 2020) and in horses in Mexico (Krebs et al. 2001; Ortega-Sánchez et al. 2022).

In Sudan, since the first laboratory confirmation of rabies in a donkey in 1961 (El Nasri 1962), rabies in horses, donkeys and mules was continuously reported (Ali 2002; Hameid 1989; Harbi 1976). During 1992–2002, of the reported rabies suspected cases, donkeys were the third most commonly affected species (Ali et al. 2006; Ali & Intisar 2009; Baraa et al. 2012) and the second most affected species in an outbreak in Darfur (Ali 2002).

Since the first laboratory identification of rabies in a donkey in Sudan in 1961 (El Nasri 1962), rabies in horses, donkeys, and mules has been consistently documented (Ali 2002; Hameid 1989, 1991; Harbi 1976). Donkeys were the third most affected species among reported rabies cases between 1992 and 2002 (Ali et al. 2006; Khalafalla & Ali 2022). This article investigates the current situation of equid rabies in Sudan through analysis of the monthly and annual reports of the Ministry of Animal Resources and fisheries during 2010–2022, including the detection of rabies virus antigen and nucleic acid in brain samples submitted to the Central Veterinary Research Laboratory for the laboratory diagnosis of suspected rabid animals.

## Research methods and design

### Collection of data

**FIGURE 1:**
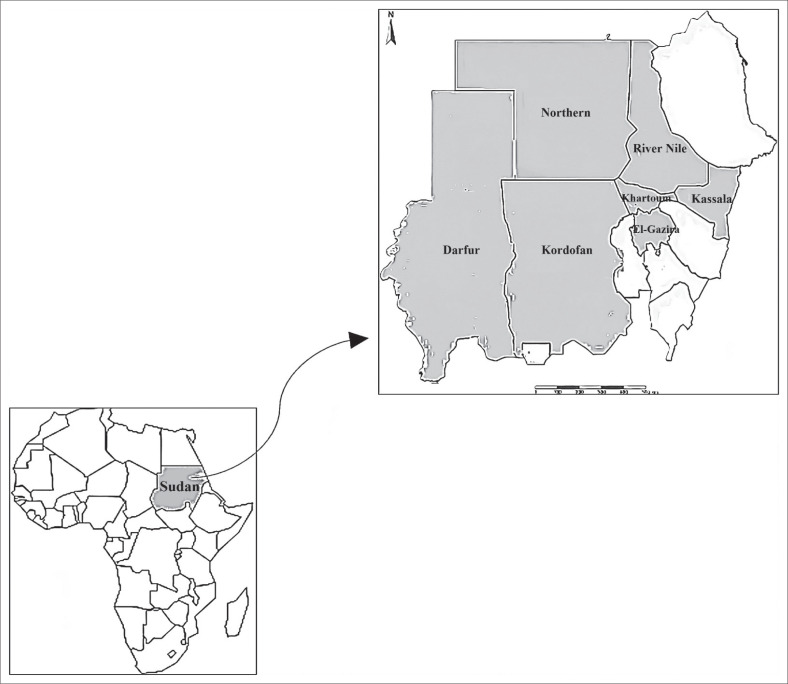
Map showing the geographic distribution of equid rabies outbreaks (*n* = 35) reported during 2010–2022 in Sudan.

Data about the occurrence of equid rabies in Sudan that occurred during 2010–2022, including the reported outbreaks, affected animal species and actions taken by the local veterinary authorities including vaccination, slaughter and destruction of clinically suspected animals were undertaken by the Animal Health Sector of the Ministry of Animal Resources and Fisheries. Outbreaks were reported in Northern, River Nile, Khartoum, El Gazira Kassala, Kordofan and Darfur States (Figure 1).

### Statistical analysis

The data were documented, coded, and saved in a Microsoft Excel spreadsheet. It was transferred to IBM^®^ SPSS^®^ software version 27. The data were summarised using descriptive and analytical statistics as needed. Year, season, state, death and the government’s response, which included vaccination and destruction of the affected animals, were the units of study. For every category of likely risk factor, the proportion of rabid cases was calculated by dividing the total number of animals registered by the number of animals that were suspected clinical cases.

### Pearson Chi-square

Pearson Chi-square test was performed to determine the correlation between clinically suspected equids and variables recorded. Statistical significance was determined at *p* < 0.05, with a 95% confidence level for the analyses.

### Logistic regression analysis

Binary logistic regression was used to find the best-fitted model to understand the correlation between rabid animals and year, season and state.

### Model coefficients

The omnibus test is used to discuss the model’s goodness of fit; we use the likelihood ratio that follows the Chi-square distribution. The Hosmer–Lemeshow test was used to test the null hypothesis, which states that the model’s predictions agree exactly with the observed group membership (Saad, Adam & Abdelateef 2016). A Chi-square statistic is produced by comparing the observed frequencies to those predicted by the linear model. Statistical significance was set at *p* < 0.05, with a 95% confidence level for the analyses.

### Laboratory diagnosis

#### Collection of samples

Brain tissues were collected from clinically suspected rabid animals (*n* = 36), aseptically handled, put on ice and submitted to the Rabies Unit, Central Veterinary Research Laboratory, Khartoum.

#### Fluorescent antibody test

Samples (*n* = 36) were tested for the detection of rabies antigen using the fluorescent antibody test (FAT), as previously described (Dean & Abelseth 1973).

#### Extraction of viral ribonucleic acid

Brain tissues collected from clinically suspected rabid donkeys and horses were used for extraction of total ribonucleic acid (RNA) (Table 1). TRIzol kits were utilised according to the manufacturer’s instructions (Gibco BRL). Simultaneously, a brain collected from uninfected mice was included as a negative control.

**TABLE 1:** Tissues collected from clinically rabid equids for ribonucleic acid (RNA) extraction.

Tissues	Animal species	State
Brain	Donkey	Khartoum
Brain	Donkey	River Nile
Brain	Horse	Khartoum
Brain	Horse	El Gazira
Brain	Horse	Darfur

#### Complementary deoxyribonucleic acid synthesis

The complementary deoxyribonucleic acid (cDNA) was synthesised using the Transcriptor First Strand cDNA synthesis Kit (Roch, Inc.) according to the protocol provided. Briefly, 1 µL random hexanucleotide primers (600 pmol/µL), 4 µL 5× reaction buffer (8 mM MgCl2), 0.5 µL RNase inhibitor (40 U/µL), 0.5 µL reverse transcriptase (20 U/µL), 2 µL 10 Mm dNTP mix and 7 µL nuclease free water were mixed with 5 µL of RNA.

### Reverse transcriptase polymerase chain reaction

Amplification of 5 mL cDNA was conducted following the procedure described by Heaton et al. (1997). Briefly, 50 µL reaction mix was prepared as follows: polymerase chain reaction (PCR) buffer containing 1.5 mM MgCl_2_, 200 mM dNTP, 2.5 pmol of each primer (JW10; GTCATTAGAGTATGGTGTTC and JW12; ATGTAACACCCCTACAATTG) and 0.5 U of *Taq polymerase* (Invitrogen). Cycling was implemented as follows: heating at 95 °C for 10 min, cycled five times at 95 °C for 90 s, 45 °C for 90 s, 50 °C for 20 s and 72 °C for 90 s and then 25 times at 95 °C for 30 s, 45 °C for 60 s, 50 °C for 20 s and 72 °C for 60 s. The final cycle was of 95 °C for 30 s, 45 °C for 90 s and 50 °C for 20 s, with a final extension at 72 °C for 10 min. The expected amplicons (~586 base pair [bp]) were documented using ethidium bromide-stained gel electrophoresis.

### Ethical considerations

Ethical clearance to conduct this study was obtained from the Central Veterinary Research Laboratory (CVRL), Sudun.

## Results

### Frequency of equine rabies

**FIGURE 2:**
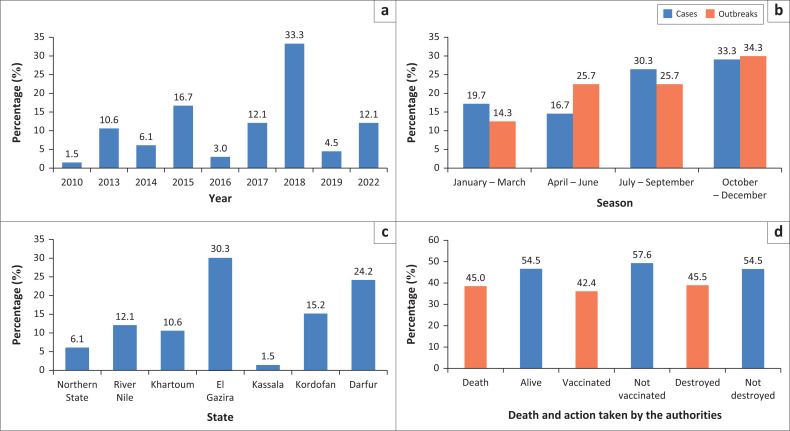
Percentage of clinically suspected rabid animals according to year (a), season (b), state (c), death, vaccination and destruction by the veterinary authorities (d).

During the period of the study, 35 equid rabies outbreaks were reported, with 66 animals affected. The highest reported incidence was seen in Al Gezira (30.3%), followed by Darfur (24.2%) and Kordofan (15.2%) states (Figure 2 and Table 2). Within years, the highest incidence rate was observed during 2018 (33.3%), followed by 2015 (16.7%) as shown in Figure 2.

**TABLE 2:** Percentage of clinically suspected equid rabies according to the year, location and season.

Variable	Clinically suspected	
	*n*	% of total
**Year**		
2010	1	1.5
2013	7	10.6
2014	4	6.1
2015	11	16.7
2016	2	3.0
2017	8	12.1
2018	22	33.3
2019	3	4.5
2022	8	12.1
Total	66	100.0
**State**		
Northern State	4	6.1
River Nile	8	12.1
Khartoum	7	10.6
El Gazira	20	30.3
Kassala	1	1.5
Kordofan	10	15.2
Darfur	16	24.2
Total	66	100.0
**Season**		
January – March	13	19.7
April – June	11	16.7
July – September	20	30.3
October – December	22	33.3
Total	66	100.0

### Seasonality of rabies

**FIGURE 3:**
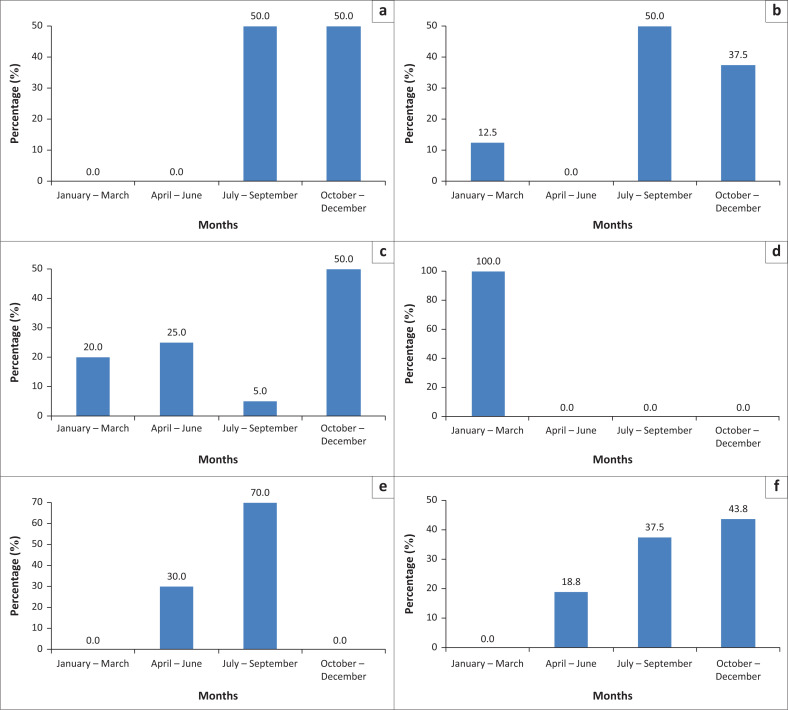
Seasonality of equid rabies cases in Northern State (a), River Nile (b), El Gezira (c), Khartoum and Kassala (d), Kordofan (e) and Darfur (f) during 2010–2022.

Statistical analysis of the data collected during the period of the study revealed that the highest percentage of the outbreaks (34.3%) as well as cases (33.3%) were reported during October – December followed by July – September (30.3%, 25.7%) (Figure 2). However, it was noticed that most of the outbreaks as well as cases were different in different states, but for Al Gezira and Darfur states have been reported during July – September and January – March. The highest reported prevalence from July to September (70%) was in Kordofan state (Figure 3).

### Decisions taken by the veterinary authorities

**FIGURE 4:**
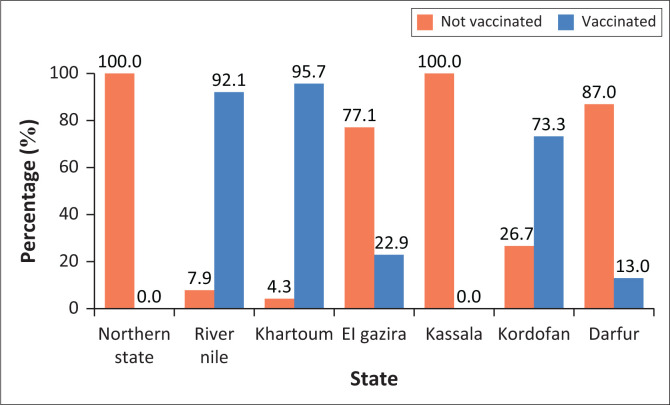
Coverage of vaccination in response to the equid rabies outbreaks according to states investigated.

Rabid animals either died of the disease (45%) or were destroyed (45.5%). Vaccination rates in response to the outbreaks varied by state, with Khartoum reporting the highest rate (95.7%), followed by 92% in River Nile State (Figure 2 and Figure 4).

### Pearson Chi-square

Pearson Chi-square analysis indicated a significant association between clinically rabid animals, year (*p* < 0.001) and state (*p* < 0.001) (Table 3).

**TABLE 3:** Pearson Chi-square analysis of clinically suspected rabies according to year, season and state.

Parameter	Year	Season	State
Pearson Chi-square	273.80^a^	159.94a	109.52^a^
*df*	8	3	6
Asymptotic significance	< 0.001	< 0.001	< 0.001

*df*, degree of freedom.

a , Each table the Chi-square test has expected a count less than 5 without affecting the accuracy of the test.

### Multivariate analysis

Omnibus test confirms the significance of the fully successful model (*p* = 0.000). The Hosmer and Lemeshow test indicates that the data fit the model (*p* = 0.772) (Table 4). The observed and predicted groups revealed that all cases were classified as in-contact, while 81.8% were correctly classified as clinically suspected rabies. The overall percentage indicates a good fit of the model.

**TABLE 4:** Statistical significance of variables included in the fitted mode for the occurrence of equid rabies.

Step 1†	B	s.e.	Wald	*df*	Sig.	Exp(B)	95% CI for Exp(B)	
							Lower	Upper
Year	0.218	0.046	21.98	1	0.000	1.25	1.14	1.37
Season	−0.609	0.205	8.84	1	0.003	0.544	0.364	0.813
State	−0.352	0.062	31.67	1	0.000	0.704	0.622	0.795
Constant	−439.77	93.58	22.09	1	0.000	0.000	-	-

s.e., standard error; *df*, degree of freedom; Sig., significance; CI, confidence interval.

† , Variable(s) entered on step 1: Year, Season, State.

From Table 3 we can fit the logistic regression model as:


$$\begin{array}{l} \text{Log}(\text{odds})=-439.761+0.218*\text{Year}-0.609\\ {}*\text{Season}-0.352*\text{State} \end{array}$$ [Eqn 1]

Wald statistics showed that year (*p* = 0.000), season (*p* = 0.003) and state (*p* = 0.000) correlates significantly with rabies virus infection.

### Laboratory diagnosis

**FIGURE 5:**
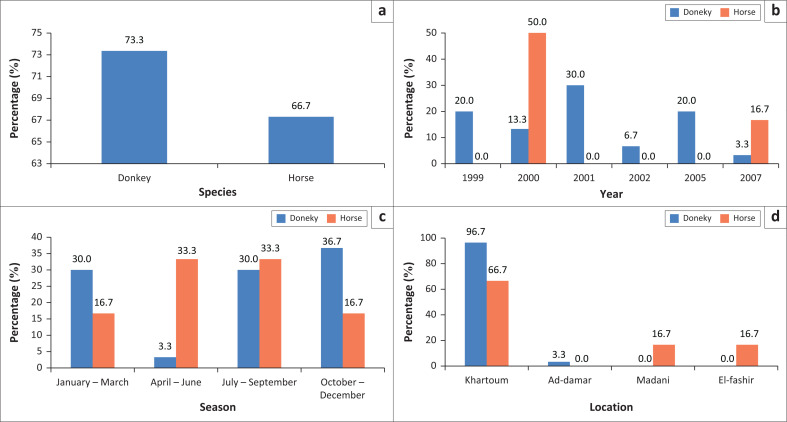
Percentage of positive equid rabies cases as confirmed by fluorescent antibody test among species (a), year (b), season (c) and location (d).

During 1999–2015, 36 equid brain samples were examined for rabies antigen using FAT. Twenty six samples tested positive (72.2%), of which 22 were donkeys (84.6%) and 4 horses (15.4%). Most of the submitted samples were from Khartoum State (96% donkey and 66% horses). Most of donkey samples were collected in 2001 (30%), while most of the horse samples (50%) were collected in 2000. Statistical analysis of the data collected during the period of the study showed that as seen in reported outbreaks and cases, most of the donkey samples (36.7%) were obtained during October – December, while most of the horse samples (33.3%) were during July – September and April – June (Figure 5, Table 5).

**TABLE 5:** The results of equid rabies antigen detection with the aid of the fluorescent antibody test in Sudan during 1999–2015.

Variable	Positive		Total	
	*n*	%	*n*	%
**Species**				
Donkey	22	84.6	30	83.3
Horse	4	15.4	6	16.7
Total	26	100.0	36	100.0
**Year**				
1999	4	15.4	6	16.7
2000	3	11.5	7	19.4
2001	6	23.1	9	25.0
2002	2	7.7	2	5.6
2005	5	19.2	6	16.7
2007	2	7.7	2	5.6
2009	1	3.8	1	2.8
2014	1	3.8	1	2.8
2015	2	7.7	2	5.6
Total	26	100.0	36	100.0
**Season**				
January – March	10	38.5	10	27.8
April – June	1	3.8	3	8.3
July – September	7	26.9	11	30.6
October – December	8	30.8	12	33.3
Total	26	100.0	36	100.0
**Location**				
Khartoum	23	88.5	33	91.7
Ad-damar	1	3.8	1	2.8
Madani	1	3.8	1	2.8
El-fashir	1	3.8	1	2.8
Total	26	100.0	36	100.0

### Reverse transcriptase polymerase chain reaction

**FIGURE 6:**
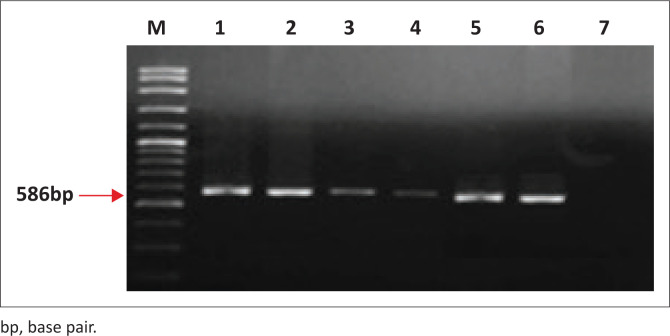
Ethidium bromide-stained agarose gel, Lane M: deoxyribonucleic acid (DNA) ladder, Lane 1–3: donkey brain, Lane 4–6: horse brain, Lane 7: negative control. The product size is 586-bp.

All tissues tested (*n* = 6) gave bands that correspond to the expected size (~586 bp). When the negative control was used as a template, no amplification product was detected (Figure 6).

## Discussion

In Sudan rabies is endemic, and since 1963, outbreaks have been continuously reported. Apart from dogs and cats, cases were reported in different animal species including equids (Ali et al. 2006; Ali, Hameid & Ibrahim 2001; Ali & Intisar 2009; Khalafalla & Ali 2022).

In the present study that represents the years 2010–2022, reported rabies outbreaks (*n* = 35) as well as the number of affected equids (*n* = 66) in Sudan is very low compared to the previous records (467 donkeys, 3 horses) during 1992–2002 (Ali et al. 2006). However, it seems to be somewhat increased compared to reported number of cases (*n* = 26) in Sudan during 2007–2010 (Baraa et al. 2012). Nonetheless, these figures are most probably less than the actual number of cases as there is a continuous problem of under-reporting, which was previously presented and highlighted in most of the rabies articles from Sudan (Ali et al. 2001, 2006) as well as Arab countries and other countries worldwide (Alaifan & Altamimi 2019; Ali et al. 2006; Bannazadeh Baghi et al. 2018; Gautret et al. 2011; Monje et al. 2021; Ripani et al. 2017; Suu-Ire et al. 2021). Rabies in equids is reported worldwide with varying incidence rates. Our results showed that the current situation of equid rabies in Sudan is in agreement with that reported in neighbouring and other African countries. In neighbouring countries, in Ethiopia, equids were found to be the second most common source of rabies infection in humans according to 27% of questionnaire responders (Digafe et al. 2015). Also rabies was considered one of the top five diseases affecting donkeys (Stringer et al. 2017); however, seven rabies cases in equids were reported in the country during 2009–2010 (Stringer et al. 2017). In Kenya, between 1958 and 2017, 113 equid rabies cases were confirmed (Bitek et al. 2018). In Nigeria (Alhassan et al. 2020) as well as in Algeria, a case of rabies in a donkey was reported (Benchohra 2016). Low incidences have been reported in Asia, in China, one case in a donkey was reported up to 2012 and another case in 2015 (Feng et al. 2016); during 2010–2020, one donkey and one horse were diagnosed (Feng et al. 2021). Equid rabies in Iran is rare; a case in a horse was reported in 2014 (Tolouei, Mobarak & Mostofi 2017). In Yemen only three rabid donkeys were reported in 2011 (Al-Shamahy, Sunhope & Al-Moyed 2013). In Mongolia, a case of a rabid horse that attacked a child, and the existence of 61 rabid horses from 2012 to 2018 was reported (Altantogtokh et al. 2023). In India, over 10 years, 13 rabid donkeys were reported (Brookes et al. 2018). The situation of equid rabies in North America is variable but still higher than that observed in our study. In United States, less than 100 cases of rabies in horses are reported annually (Kumar et al. 2018). In 2000, rabies was reported in 52 horses, donkeys and mules (Krebs et al. 2001), while during 2021, 17 horses and 1 mule were confirmed to be rabid in the United States (US) (Ma et al. 2023). Unlike Africa, Latin America usually shows the highest equids rabies incidence rates. In Brazil, equids were found to be the second most rabies-affected animals (Andrade et al. 2020). During 2010–2019, 1290 rabid horses (Oliveira et al. 2022), 571 cases between 2000 and 2003 (Carrieri et al. 2006) and during 2002–2021, 23 rabid horses were reported in Brazil (Sodré et al. 2023). In Mexico, during 2010–2019, 150 rabid horses were reported (Sodré et al. 2023). The main source of rabies in Brazil and other Latin American countries is bats; this explains the large number of cases.

During the period of the study in Sudan, the highest incidence of rabies in equids (33.3%) has been reported in 2018, followed by 2015 (16.7%). This is linked to the rabies control strategies adopted by the veterinary authorities. It is well-known that the incidence of rabies is inversely proportional to the efficient control policy adopted in Sudan (Baraa et al. 2012) and elsewhere (Nyasulu et al. 2021; Rahman et al. 2020). The picture of rabies incidence in Sudan during the study period appears to have cycles of increase every 3–6 years. This is in line with a previous report about rabies epidemics in Southern and Eastern Africa, and a similar picture was observed in Sudan, Botswana, Kenya, Namibia, South Africa and Zimbabwe (Hampson et al. 2007).

Rabies in equids as well as other species exists in different locations in Sudan; however, during the current study it was noticed that highest reported incidences were seen in Al Gezira in Central (30.3%), followed by Darfur (24.2%) and Kordofan in Western Sudan (15.2%). With the exception of Khartoum, the same situation was previously reported in Sudan (Ali et al. 2001, 2006; Ali & Intisar 2009) as these areas have the largest number of animals. The high incidence of rabies is expected to occur in more animal-dense areas, where there are more susceptible animals (Nyasulu et al. 2021; Oliveira et al. 2022; Rees et al. 2011).

In this study, the incidence of rabies in equids was observed to increase during October–December (34.3% of outbreaks and 33.3% of cases), followed by July–September (25.7% of outbreaks and 30.3% of cases). Rabies is known to occur all over the year; however incidence of the disease occurs more in some periods during the year. This seems to show some sort of seasonality, which is in line with the previous reports in Sudan (Ali et al. 2001, 2006; Ali & Intisar 2009). As equids are kept in close contact with dogs and other animals, it is expected that they will infect humans and other animals. This is linked to fighting during the mating season of dogs that are the main animals involved in rabies epidemiology (Yadav 2012) or the increase in the dog population as a consequence of new born puppies (Douangngeun et al. 2017). Seasonality of rabies is suggested and documented in different countries such as Africa, Morocco, Algeria (El Harrak 2012; Khayli et al. 2019) and Tunisia (Hassine et al. 2021). It was also reported in Asia, in Bhutan (Tenzin, Dhand & Ward 2011), India (Brookes et al. 2018) and Nepal (Pantha et al. 2020), in Latin America, Peru (Malaga, Nieto & Gambirazio 1979).

In the present study, decisions taken by the veterinary authorities in response to rabies outbreaks included destruction of animals that showed obvious clinical signs of rabies (45%) and vaccination of susceptible in-contact ones. Vaccination of equids in response to rabies outbreaks was applied in different states, with the highest figures reported in Khartoum State (95.7%), and River Nile State (92%). This may be expected because of the increased public awareness and the accessibility of vaccines in those states.

When comparing our findings using bivariate and multiple regression analysis, we might see slight variations. For example, the Chi-square test indicates that there is a significant relationship between cases and both state and destruction of animals. However, logistic regression is adopted to predict the probability of the outcome variable given the predictor variable and to identify important predictor variables in the model (Kleinbaum, Klein & Pryor 2002). In our investigation, season is predicted to contribute to the occurrence of equid rabies.

Laboratory diagnosis of rabies in Sudan is routinely applied using the FAT. The number of cases tested for rabies in this study was 36, 72.2% of which tested positive. The majority of positives (22) were donkeys (84.6%) followed by four horses (15.4%). A similar result was reported in Sudan between 2003 and 2007, where five of six donkey samples were positive (Ali and Intisar 2009). Generally, equid laboratory confirmed cases are low in Sudan as well as other African countries, which is mainly because of under-reporting and people not being interested to report rabies in animals. This was previously pointed out in different reports in Sudan (Ali et al. 2001, 2006; Ali & Intisar 2009; Hameid 1991). The same situation was observed in different countries, between 2008 and 2012, only one horse and four donkeys were diagnosed for rabies in the laboratory in Burkina Faso (Minoungou et al. 2021). In Kenya, over a period of 59 years, 113 laboratory rabies confirmed cases in equids were reported (Bitek et al. 2018). In Malawi, two donkeys were confirmed (Kainga et al. 2023). During 1993–2019, 72 confirmed rabid horses were reported in South Africa (Koeppel et al. 2022). The highest number of horse rabies confirmed cases (203) was reported during 2012–2018 in Tunisia (Kalthoum et al. 2021) and 44 during 2011–2017 in Brazil (Ribeiro et al. 2021).

In Sudan, equids are usually kept in close contact with humans and other animals, so they can easily contract the disease and be a source of infection. It is concluded that rabies in equids in Sudan is a continuous public health and economic problem and it is recommended to include equids in rabies vaccination programmes in areas with high numbers of equids.
